# Anlotinib suppressed tumor cell proliferation and migration in hypopharyngeal carcinoma

**DOI:** 10.1016/j.bjorl.2024.101397

**Published:** 2024-01-25

**Authors:** Hao Song, Qing Song, Xiangkun Zhao, Yuteng Yang, Yakui Mou, Yumei Li, Xicheng Song

**Affiliations:** a The Second Medical College, Binzhou Medical University, Yantai, China; b Department of Otorhinolaryngology Head and Neck Surgery, Yantai Yuhuangding Hospital, Qingdao University, Yantai, China; c Yantai Key Laboratory of Otorhinolaryngologic Diseases, Yantai, China; d Shandong Provincial Clinical Research Center for Otorhinolaryngologic Diseases, Yantai, China; e Department of Otorhinolaryngology Head and Neck Surgery, Yantai Yeda Hospital, Yantai, China

**Keywords:** Anlotinib, HIF-1α, Hypopharyngeal carcinoma, Migration, Proliferation

## Abstract

**Objective:**

The purpose of this study is to study the in-vitro effects of multitarget inhibitor anlotinib on hypopharyngeal cancer cell proliferation and cell migration, and the underlying mechanism, which will provide new drug choices for hypopharyngeal cancer treatment.

**Methods:**

The Hypopharyngeal cancer Fadu cells were treated with anlotinib at a concentration of 0, 5, and 10 μmoL/L, respectively. Cell counting kit-8 and the colony-forming assay were used to detect the inhibition of cell proliferation. Wound-healing assay and transwell assay were used to detect the migration and invasion ability of cells. Flow cytometry was used to detect the effects of anlotinib on cell cycle and apoptosis. RT-qPCR and Western blot were used to measure gene expression levels.

**Results:**

CCK-8 and colony-forming assay showed that anlotinib could significantly inhibit cell proliferative activity. Wound-healing assay and transwell assay showed that anlotinib could inhibit cell migration and scratch. These results showed that anlotinib has obvious antitumor activity. Flow cell cycle experiment showed that anlotinib could promote Fadu cell apoptosis and block the G2/M phase for inhibiting cell proliferation. In addition, anlotinib decreased the expression of HIF-1α.

**Conclusions:**

Anlotinib has an excellent suppressing effect on the proliferation, migration, and invasion of hypopharyngeal cancer Fadu cells in-vitro. Moreover, it can play an anti-tumor role through blocking cell cycle G2/M and promoting apoptosis, which may be related to the decrease of HIF-1a expression. Our study would provide a potential treatment method for patients with hypopharyngeal cancer.

**Level of evidence:**

Level 3.

## Highlights

• Anlotinib inhibits the proliferation of Fadu cells in hypopharyngeal carcinoma.
• Anlotinib blocks G2/M and promotes apoptosis in the hypopharyngeal cancer cell cycle.
• Anlotinib puts Fadu cells in a hypoxic state, which in turn inhibits cell proliferation.

## Introduction

The hypopharynx extends from the superior edge of the hyoid bone to the inferior aspect of the cricoid cartilage and includes three subsites: the piriform sinuses, the posterior hypopharyngeal wall, and the postcricoid area.^1^ Hypopharyngeal carcinoma is usually found in the piriform sinuses, followed by the posterior hypopharyngeal wall and the postcricoid area. The early symptoms of hypopharyngeal carcinoma are not obvious, and often were detected in advanced stages.^2^, ^3^ Surgery and radiotherapy are the main treatment methods for hypopharyngeal cancer.^4^ Surgery and radiotherapy alone can significantly improve the prognosis of patients in the early stages (I and II), but the efficacy of single treatment for patients in the advanced stages (III and IV) is poor.^2^ The combined treatment with radiotherapy, chemotherapy, and biological therapy can significantly improve the local control rate and laryngeal retention rate of tumors, which are gradually applied in the treatment of hypopharyngeal cancer. However, clinical data show that the 5-year survival rate of patients has not been significantly improved.^5^ Surgery sometimes causes hoarseness and even loss of vocal function, which seriously reduces the quality of life of patients. The higher dose of radiotherapy and chemotherapy for some patients who are intolerant to radiotherapy and chemotherapy will have serious toxic and side effects.^6^, ^7^, ^8^, ^9^ Targeted drugs have much better treatment effects, however, limited targeted drugs could be chosen for hypopharyngeal cancer treatment. Therefore, it is urgent to find a new treatment method to improve the quality of life of patients and improve the local control rate of the larynx.

Anlotinib is a multi-target tyrosine kinase inhibitor that binds to multiple receptors. Anlotinib binds to Vascular Endothelial Growth Factor Receptors 1, 2, and 3 (VEGFR 1, 2, and 3) and inhibits the activation of downstream signaling pathways to regulate tumor microenvironment.^10^ Anlotinib can inhibit the phosphorylation expression of related proteins and induce apoptosis of tumor cells by acting on Fibroblast Growth Factor Receptor (FGFR), Platelet-Derived Growth Factor Receptor (PDGFR-β) and downstream Extracellular Regulated protein Kinase (AKT/ERK) signaling pathway, in a dose-dependent manner.^11^, ^12^, ^13^ The formation of abnormal blood vessels in the tumor microenvironment is closely related to tumor genesis and development. Both the receptors VEGFR and FGFR and their ligands VEGF and PDGF are highly expressed in head and neck tumors and the high expression of PDGF can promote the expression of VEGF.^14^, ^15^ The high expression of VEGF plays an important role in the formation of tumor blood vessels and the growth of tumor cells.^10^ As a multi-target inhibitor, anlotinib can inhibit both VEGFR and PDGFR, thus significantly inhibiting the formation of tumor blood vessels and tumor growth.^16^, ^17^

Hypoxia is an independent adverse factor affecting tumor prognosis. Hypoxia-Inducible Factor (HIF-1α) is highly expressed in a variety of tumors, and its expression level is correlated with tumor stage and prognosis.^18^, ^19^ The formation of abnormal blood vessels in the tumor area and the change in the permeability of the blood vessel wall caused the tumor cells to be in a hypoxic state. Anlotinib improves the local oxygenation of tumors by reducing abnormal angiogenesis and inducing the normalization of abnormal blood vessels. Meanwhile, it can reduce tumor interstitial fluid pressure, increase drug penetration in tumors, and avoid hypoxia-induced immune escape.^16^, ^17^ Gao et al.^20^ found that anlotinib-treated lung cancer mouse models showed reduced HIF-1α expression. So, we assume that anlotinib may inhibit the tumor by regulating HIF-1α expression. In this study, we explored the inhibitory effect of anlotinib on hypopharyngeal cancer cells and explored the possible mechanism of action in vitro, which will potentially provide a new choice for the treatment of hypopharyngeal cancer.

## Methods

### Cell lines and cell culture

Hypopharyngeal squamous cell line Fadu cell was obtained from Cell Bank, Chinese Academy of Sciences. The cell lines were cultured in DMEM medium with 10% Fetal Bovine Serum (FBS) and 0.1% Penicillin streptomycin.

### Cell counting kit-8 (CCK-8)

Cells (6 × 10^3^/well) treated by 0, 5, 10 μmoL/L anlotinib (MedChemExpress, China) were incubated with 10 u L cck-8 (Sparkjade, China) for 1 h. The absorbance OD values of each well at 24, 48, and 72 h were measured at the wavelength of 450 nm. Each group included 6-replicate wells.

### Colony-forming assay

One thousand Fadu cells per well were inoculated into 6-well plates with a 2 mL cell culture medium. The cell was placed in 37 °C 5% CO_2_ for continuous culture and the medium was changed every three days until obvious colony formation was found in 6-well plates. Then the cells were fixed with methanol for 30 min and stained with Giemsa. The number of colonies in each well was calculated and analyzed statistically.

### Wound-healing assay

Fadu cells were seeded into 6-well plates with a cell density of 6 × 10^5^ cells/well. After the cells reached 90% confluence, a 200 μL pipette tip and ruler were used to slide perpendicular to the plate to create cell cracks. Serum-free medium was added for culture 0 and 48 h. Migration distance was photographed and calculated using Image J software.

### Transwell assay

The matrigel was melted at 4 °C and diluted with the serum-free medium at a ratio of 1:6. The Transwell chamber, was incubated in 37 °C environments for 1 h, after that, 50 μL diluting matrigel was added. After the matrigel was solidified, 800 μL DMEM medium containing 20% FBS was added to the lower chamber, and 1 × 10^4^ cells were inoculated in the upper chamber and incubated for 48 h. Methanol was used to fix the transwell chamber for 30 min and Giemsa was used to stain it for 30 min. Five microscopic fields were randomly selected and the number of invaded cells in each field was calculated for statistical analysis.

### Cell cycle and apoptosis assays

Logarithmic growth cells were inoculated on 6-well plates. Three parallel samples were set in each group. Fadu cells were treated with different concentrations of anlotinib (5, 10 μmoL/L) for 48 h. 5 × 10^5^ cells in each group were collected by digestion and centrifugation and then were processed according to the instructions of the cell cycle assay kit (Elabscience, China). Finally, the cell cycle was detected by flow cytometry. The cells were digested with trypsin without EDTA and centrifuged at 300 g for 5 min for 4 °C. Pre-cooled PBS was used to wash cells and 1 × 10^5^ cells were collected. Before Annexin V-FITC (BECKMAN, USA) and propidium iodide were used to stain cells, the cell precipitate was re-suspended with binding buffers. The samples were determined by flow cytometry.

### Real-time quantitative polymerase chain reaction (RT-qPCR)

Cell precipitation was collected, total RNA was extracted with Trizol reagent (Ambion, Austin, Texas, USA), and RNA concentration was measured. The extracted RNA was reversely transcribed into cDNA according to the instructions of the reverse transcription kit (AG, China) and then stored at −20 °C (SparkJade, China). 2^−△△Ct^ value denotes the relative expression level of mRNA. The primer sequences for RT-qPCR are shown in Table 1.

**Table 1:** qPCR primer sequences for genes.

Primer name	Primer sequence (5’-3’)
GAPDH	Forward primer TGTTGCCATCAATGACCCCT
	Reverse primer CTCCACGACGTATCAGCG
HIF-1α	Forward primer AAAATCTCATCCAAGAAGCC
	Reverse primer AATGTTCCAATTCCTACTGC

### Western blot

The protein was extracted from cells with RIPA Lysis Buffer (Solarbio, China). The protein concentration was measured with an enhanced BCA protein assay kit (Beyotime, China). After that, the protein was added to the loading buffer, then was boiled at 100 °C for 10 min and stored at −20 °C. The proteins were isolated by 8% sodium dodecyl Sulfate-Polyacrylamide Gel Electrophoresis (SDS-PAGE) and then transferred to a Polyvinylidene Difluoride (PVDF) membrane. The PVDF membrane was sealed with 5% skim milk for 2-hs. After the sealing, the primary antibody was incubated at 4 °C overnight. The membrane was washed and then incubated in the secondary antibody for 2-hs. After incubation, the membrane was washed with TBST again. The protein bands were visualized with a developer and then analyzed with Image J software.

### Statistical analysis

The data were analyzed by SPSS 16.0 software. Comparison between groups was performed by one-way ANOVA. All values were reported as mean ± SD and analyzed by Student's *t*-test or one-way analysis of variance; *p-*value of < 0.05 means statistically significant.

## Results

### Anlotinib inhibits the proliferation of hypopharyngeal cancer cells

**Fig. 1:**
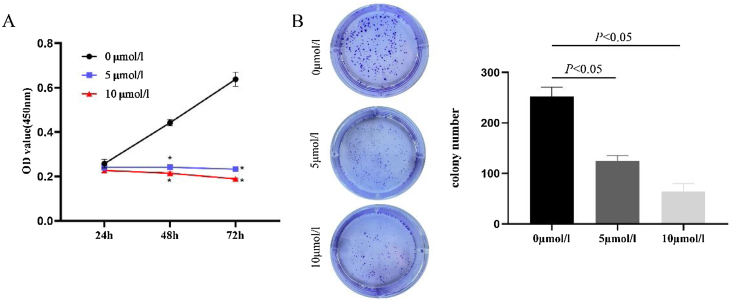
Anlotinib inhibited Hypopharyngeal cancer cell proliferation (A) Anlotinib inhibited the proliferation of tumor cells with the concentration and time increase by CCK-8 assay. (B) Through colony-forming assay, the number of Fadu cell colonies was tested and analyzed in the different concentrations of anlotinib.

In order to evaluate the effect of anlotinib on cell proliferation activity, we treated cells with anlotinib at different concentrations (0, 5, 10 μmoL/L) for 24 h, 48 h, an 72 h respectively, and then detected tumor cell viability by CCK-8. As shown in Fig. 1A, the absorbance of cells in the experimental group decreased significantly compared with the control group. At 72 h, the absorbance was highest in the control group and lowest in the 10 μmoL/L anlotinib group. The results showed that anlotinib inhibited the proliferation of tumor cells with the concentration and time increase (Fig. 1A). Through colony-forming assay, we observed that anlotinib reduced the number of cloning of Fadu cells, and with the increase of the concentration of anlotinib, the number of colonies decreased significantly (Fig. 1B). Statistical analysis showed colony formation was significantly different between the experimental and control groups (Fig. 1B). These results suggested that anlotinib could significantly inhibit the growth of Fadu cells.

### Anlotinib inhibits hypopharyngeal cancer cell migration and invasion

**Fig. 2:**
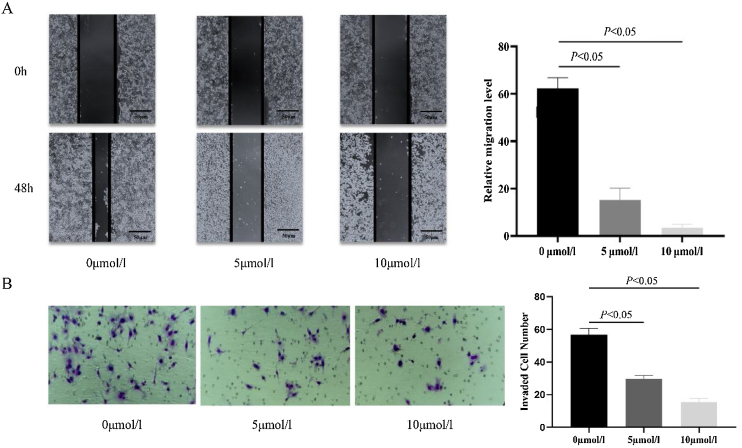
Anlotinib inhibited Hypopharyngeal cancer cell migration and invasion. (A) The cell migration distance of the control and anlotinib treatment group for 48 h by wound-healing experiment (*p* < 0.05). (B) Fadu cell invasion was measured in control and anlotinib treatment groups by transwell assays (*p* < 0.05).

In order to investigate the effect of anlotinib on Fadu cell migration, we carried out the wound-healing experiment. The migration of cells treated with different concentrations of anlotinib for 48 h was recorded. In the beginning, the wound widths between the anlotinib treatment group and the control group were roughly equal. After 48 h of treatment, the cell migration distance in the control group was significantly longer than that in the anlotinib treatment group (Fig. 2A). And the cell migration distance in the 5 μmoL/L anlotinib treatment group was significantly longer than that in the 10 μmoL/L anlotinib treatment group (Fig. 2A). These results showed that the migration of tumor cells was significantly inhibited by anlotinib in a concentration-dependent manner.

In addition, a transwell assay was performed to observe the effect of anlotinib on Fadu cell invasion. We found that when the cells were treated with 5 μmoL/L and 10 μmoL/L anlotinib for 48 h, the number of cells penetrating the matrix glue and the compartment membrane was significantly reduced compared with the control group (Fig. 2B). The study found that anlotinib inhibited the invasion ability of Fadu cells.

### Anlotinib induced G2/M arrest and increased hypopharyngeal cancer cell apoptosis

**Fig. 3:**
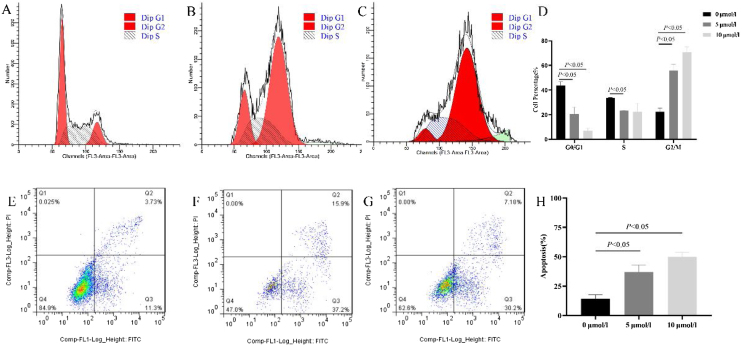
Anlotinib regulated Fadu cell cycles and promoted apoptosis in vitro. (A–D) The cell cycle was tested by flow cytometric analysis in control and anlotinib treatment groups (*p* < 0.05). (E–H) The Fadu cell death was tested by flow cytometry in control and anlotinib treatment groups (*p* < 0.05).

Previous studies showed anlotinib could significantly inhibit cell growth, however, the mechanism is not clear. Therefore, we tested anlotinib’s effect on the cell cycle and cell apoptosis. As shown in Fig. 3, compared with the control group, the number of G2/M phase cells in anlotinib treatment group increased significantly, and the number of G0/M phase cells decreased. Further analysis showed that when anlotinib concentration was 10 μmoL/L, it could intervene in the cell DNA synthesis phase (S phase) and reduce cells in the S phase. These results showed that anlotinib induced cell cycle transition from the G0/G1 phase to the G2/M phase (Fig. 3A‒D). As shown in Fig. 3E–G, the proportion of apoptotic cells in Q2 and Q3 were significantly increased in the anlotinib treatment groups compared with the control group, showing that anlotinib could promote cell apoptosis (Fig. 3H). These experiments suggested that anlotinib may inhibit cell proliferation by regulating the cell cycle and apoptosis.

### Anlotinib inhibited the expression of HIF-1α

**Fig. 4:**
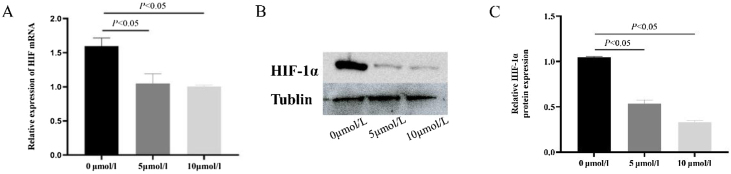
Anlotinib inhibited the expression of HIF-1α in hypopharyngeal carcinoma cells. (A) The expression of HIF-1α mRNA in Fadu cells of hypopharyngeal carcinoma treated with anlotinib was tested by RT-qPCR (*p* < 0.05); (B) HIF-1α expression in control and anlotinib treatment groups was tested by western blot and statistically analyzed (*p* < 0.05).

After 5 μmoL/L and 10 μmoL/L anlotinib treatment, the expression of HIF-1α in tumor cells was detected by qPCR (Fig. 4A) and western blot (Fig. 4B and C). It was found that compared with the control group, 10 μmoL/L anlotinib could significantly reduce the expression of HIF-1α, which showed that anlotinib could inhibit the expression of HIF-1α.

## Discussion

In recent years, targeted drugs have gradually become the focus of scholars due to their advantages of strong specificity, significant curative effect, and convenient use.^21^ However, targeted drugs used in head and neck cancer treatment are relatively limited. cetuximab is the mainly targeted drug in the treatment of head and neck cancer, however, studies have found that cetuximab can cause severe skin and nail toxicity in some patients and may interrupt treatment.^21^ So, the development of new targeted drugs will benefit the treatment of head and neck cancer. Anlotinib can play an anticancer role in a variety of tumors by targeting VEGF, FGFR, PDGFR, and other receptors, but whether it can treat head and neck tumors remains unclear.^21^ Our study found that anlotinib inhibited the proliferation and migration, and induced cell cycle arrest and apoptosis of hypopharyngeal cancer cells, thus inhibiting tumor growth. Our study may provide new drug options for the treatment of head and neck tumors. At the same time, our study found that anlotinib can inhibit HIF-1α expression. It suggested that anlotinib may inhibit the proliferation of hypopharyngeal cancer cells by regulating HIF-1α, which provides a new research direction for the mechanism of anlotinib.

Anlotinib, a multi-target tyrosine kinase inhibitor, was reported to have antitumor activity by experimental studies and clinical trials in a variety of tumors like colorectal cancer soft tissue sarcoma, and non-small cell lung cancer.^10^, ^22^, ^23^ It can inhibit the proliferation and invasion of tumor cells by acting on VEGFR, FGFR, PDGFR-β, and downstream AKT, ERK.^9^, ^13^ Studies have found that anlotinib regulates the cell cycle and induces cell cycle arrest by inhibiting FGFR, PDGFR, PI3K, and its downstream AKT, ERK signaling pathway, and the phosphorylation of corresponding proteins.^11^, ^12^, ^13^ As in other tumors, anlotinib can block hypopharyngeal cancer cells' G2/M cell cycle and reduce the G0/G1 phase cell number. In addition, the researchers found that anlotinib could interfere with the apoptosis process of tumor cells by promoting cell apoptosis by acting on VERFG-2 and inhibiting its phosphorylation. Some studies also found that anlotinib could induce the apoptosis of oral squamous cancer cells by upregulation of Bax, caspase-3, and Poly ADP-Ribose Polymerase (PAPR). Consistent with the above studies, anlotinib can promote apoptosis of hypopharyngeal cancer cells. In addition, anlotinib can regulate the local immune microenvironment of tumors. Yang et al.^24^ found that anlotinib can inhibit tumor cell growth by increasing local immune cell infiltration of the tumor. By inhibiting the expression of PD-L1 on endothelial cells, anlotinib can promote the entry of CD8 + T cells, increase the secretion of cytokines such as tumor necrosis factor α in the microenvironment, inhibit the secretion of interleukin, and increase the local immune response.^17^, ^25^

HIF-1α is a factor closely associated with oxygenation in the local microenvironment. HIF-1α can enable hypopharyngeal cancer tumor cells to tolerate the hypoxia microenvironment and can improve the ability of tumor metastasis by regulating local glucose metabolism, angiogenesis tumor invasion, and other activities.^26^, ^27^, ^28^ However, whether anlotinib could regulate HIF-1α expression remains unclear. In our experiments, we found that anlotinib regulated the production of HIF-1α. Therefore, we suspected that anlotinib could regulate tumor cell function by acting on HIF-1α, thereby affecting cell proliferation, angiogenesis, and survival. However, it still needs further experiments to verify the regulation mechanism. Our study provides a potential study direction for anlotinib on HIF-1a regulation.

In this study, we found that anlotinib could regulate the cycle and apoptosis process of hypopharyngeal cancer cells, thus inhibiting the proliferation, migration, and invasion of hypopharyngeal cancer cells. Meanwhile, we also found that anlotinib could regulate the expression of HIF-1α in hypopharyngeal cancer cells. There are still some limitations to our study. No HIF-1α high expression cell lines were further constructed for retrospective study, and only the changes of HIF-1α expression in hypopharyngeal cancer cells treated with anlotinib were studied. In the future, we will further investigate whether anlotinib can exert an inhibitory effect on hypopharyngeal cancer by directly targeting HIF-1α and regulating the expression of HIF-1α by changing local oxygen.

## Conclusions

Anlotinib has an excellent suppressing effect on the proliferation, migration, and invasion of hypopharyngeal carcinoma Fadu cells in-vitro. Moreover, it can play an anti-tumor role by blocking cell cycle G2/M and promoting apoptosis, which may be related to the decrease of HIF-1a expression.

## Data availability statement

The raw data supporting the conclusions of this article will be made available by the authors, without undue reservation.

## Authors’ contributions

HS and QS conceived the paper and drafted the manuscript. HS and XZ performed the experiment and drew the figures. YY, FS and YM analyzed the data. YL and XS revised the manuscript. All authors contributed to the article and approved the submitted version.

## Funding

This work was supported by the Taishan Scholars Project (nº ts20190991) and Key research and development program of Shandong (2022CXPT023).

## Statement

No human subjects or animals were involved in this study and there were no ethics issues.

## Conflicts of interest

The authors declare no conflicts of interest.
